# Cardiac Amyloidosis: A Rare Cause of Recurrent Chylothorax

**DOI:** 10.7759/cureus.24861

**Published:** 2022-05-09

**Authors:** Raj Patel, Zunairah Shah, IDO Santo, Faiz Anwer

**Affiliations:** 1 Department of Medical Education, Weiss Memorial Hospital, Chicago, USA; 2 Department of Hematology and Oncology, Cleveland Clinic, Cleveland, USA

**Keywords:** immunoglobulin light chain amyloidosis, cardiac amyloid, al amyloid, heart failure with preserved ejection fraction, chylothorax

## Abstract

Amyloidosis is caused by the extracellular deposition of fibrils composed of low molecular weight protein subunits. Its two main types are amyloid light chain (AL) amyloidosis and amyloid A (AA) amyloidosis (previously referred to as secondary amyloidosis). Clinical manifestations are dependent upon location, type, and amount of deposit.

We report a case of a 72-year-old female who presented to the emergency department for evaluation of cough, fatigue, and shortness of breath. Physical examination was notable for decreased breath sounds bilaterally over the lower lung fields. Computed tomography (CT) of the chest showed a large lobulated right pleural effusion and atelectasis. Two liters of milky, white pleural fluid was removed via thoracocentesis, and pleural fluid analysis was consistent with chylothorax. Over the next 10-day period, there was a reaccumulation of the pleural fluid, which required a pleural catheter placement. The patient underwent video-assisted thoracoscopic surgery with pleural and pericardial tissue biopsy that was consistent with kappa or lambda AL amyloid. Unfortunately, her respiratory status subsequently declined, requiring mechanical ventilation, and eventually leading to cardiac arrest.

Cardiac amyloidosis can rarely cause chylous ascites and chylothorax. The absence of electrocardiographic findings of left ventricular hypertrophy combined with apparent left ventricular hypertrophy on echocardiography is strongly suggestive of infiltrative cardiomyopathy such as cardiac amyloidosis. In these cases, cardiac amyloidosis should be considered in the differential diagnosis of chylothorax.

## Introduction

Systemic amyloidosis is a term used to describe a group of rare diseases caused by the deposition of misfolded protein fibrils (amyloid). The type and severity of the disease are dependent upon the organ involved and the type of misfolded protein [1]. In cardiac amyloidosis, these deposits build up in the heart’s extracellular space. The most common forms of cardiac amyloidosis are transthyretin and light chain amyloidosis [2].

Due to a lack of suspicion on the part of many clinicians, this condition remains largely underdiagnosed or is diagnosed only in its advanced stages. Given its high mortality and poor prognosis, early detection is of utmost importance to prevent the irreversible changes associated with advanced disease [3].

In chylothorax, chyle leaks from the lymphatic system and accumulates in the pleural space. Causes of chylothorax can be divided among traumatic and non-traumatic, cardiac amyloidosis being an uncommon subtype of the latest [4]. In this case report, we describe a case of amyloid light chain (AL) cardiac amyloidosis presenting as recurrent chylothorax.

## Case presentation

A 72-year-old African American female presented for evaluation of cough, fatigue, and shortness of breath of five days duration. Her past medical history was remarkable for coronary artery disease, hypertension, chronic obstructive pulmonary disease, obesity, and type 2 diabetes mellitus. A review of systems was significant for dyspnea with minimal exertion (New York Heart Association, Class III), lower extremity edema, and decreased appetite without significant weight loss.

**Figure 1:**
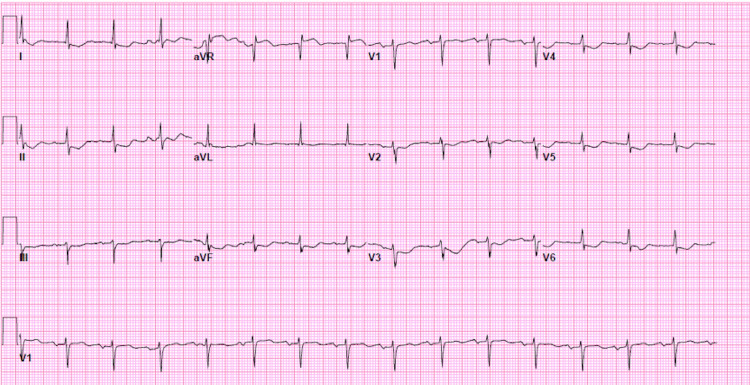
Sinus rhythm with non-specific ST-T wave abnormalities

**Figure 2:**
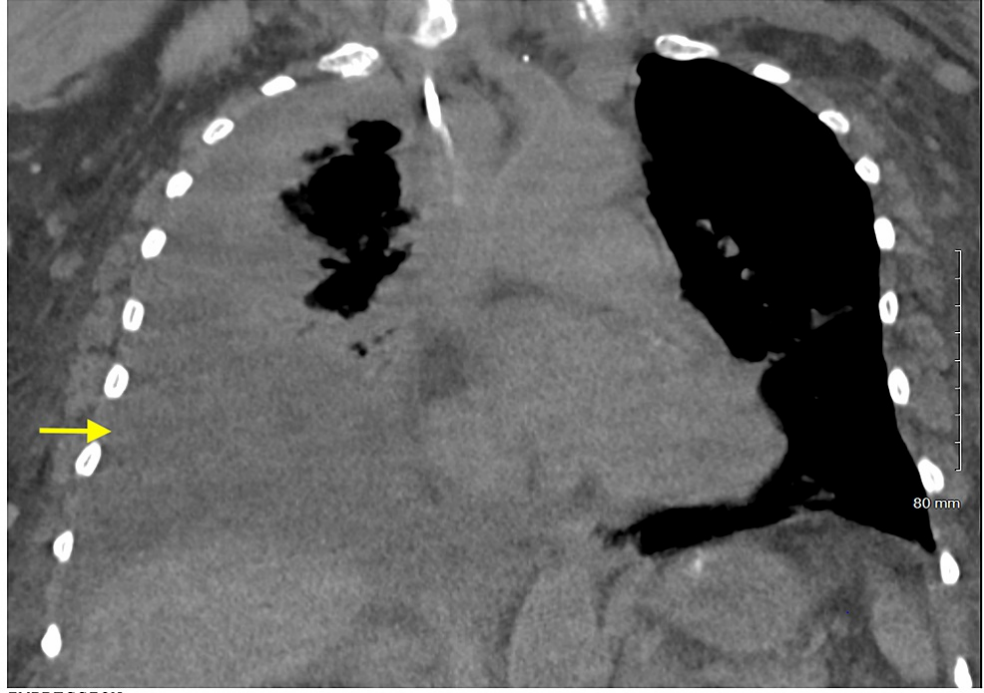
CT scan showing large right pleural effusion

On presentation, the patient was afebrile, her blood pressure was 151/62 mmHg, heart rate was 74 beats/minute, respiratory rate was 18 breaths/minute, and oxygen saturation was 93% on room air. Breath sounds were decreased bilaterally over the lower lung fields, and heart rhythm was regular without murmurs or gallops. The abdomen was firm and nontender and there was bilateral 1+ pedal edema. Electrocardiogram indicated sinus rhythm with non-specific ST-T wave changes (Figure 1). Laboratory studies are reported in Table 1. CT of the chest showed a large lobulated right pleural effusion and atelectasis (Figure 2). Two liters of milky white fluid was removed via thoracentesis, and pleural fluid analysis was significant for a triglyceride concentration of 247 mg/dl, thereby confirming the diagnosis of chylothorax.

**Table 1:** Laboratory data

Labs	Value (range)
Hemoglobin	8.3 (12.0 to 16.0 g/dL)
White blood cell count	4.6 L (4.8 to 10.8 K/UL)
Platelets	180 (150 to 450 K/L)
International normalized ratio	1.3 (0.9 to 1.1)
Creatinine	0.87 (0.7 to 1.5 mg/dL)
Sodium	144 (134 to 148 mmol/L)
Potassium	5.2 (3.5 to 5.5 mmol/L)
Total bilirubin	0.5 (0.2 to 1.4 mg/dL)
Aspartate aminotransferase	37 (0 to 40 IU/L)
Alkaline phosphatase	131 (80 to 150 U/L)
Albumin	3.3 (3.2 to 5.5 g/dL)
Total protein	5.4 (6.0 to 8.3 g/dL)
Troponin I	<0.03 (0.00 to 0.05 ng/L)
B-type natriuretic peptide	157.4 (0 to 100 pg/mL)

Over the next three months, the patient required five thoracenteses for recurrent chylothorax of unclear etiology. Fluid cytology was negative for malignancy. A cardiac echocardiogram revealed concentric left ventricle hypertrophy suggestive of infiltrative heart disease with normal systolic function and grade I diastolic dysfunction. Given the persistent reaccumulation of pleural fluid and resulting dyspnea, a decision was made to place a pleural catheter and 550-850 ml of milky fluid was drained daily to try to achieve spontaneous pleurodesis. However, after over 10 days of drainage, the patient's condition failed to improve and she was placed on mechanical ventilation due to worsening respiratory status.

**Figure 3:**
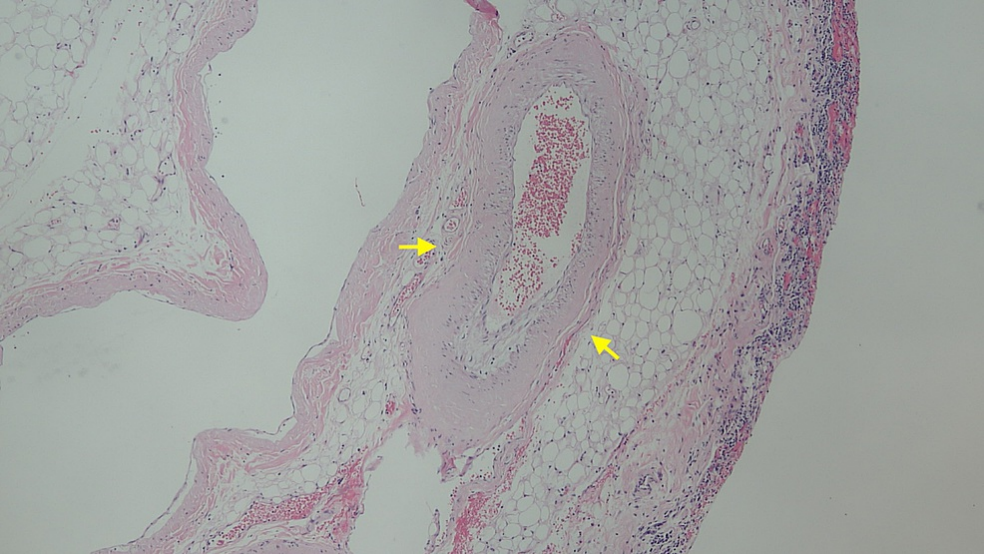
Perivascular homogeneous deposits within the walls showing amyloidosis

**Figure 4:**
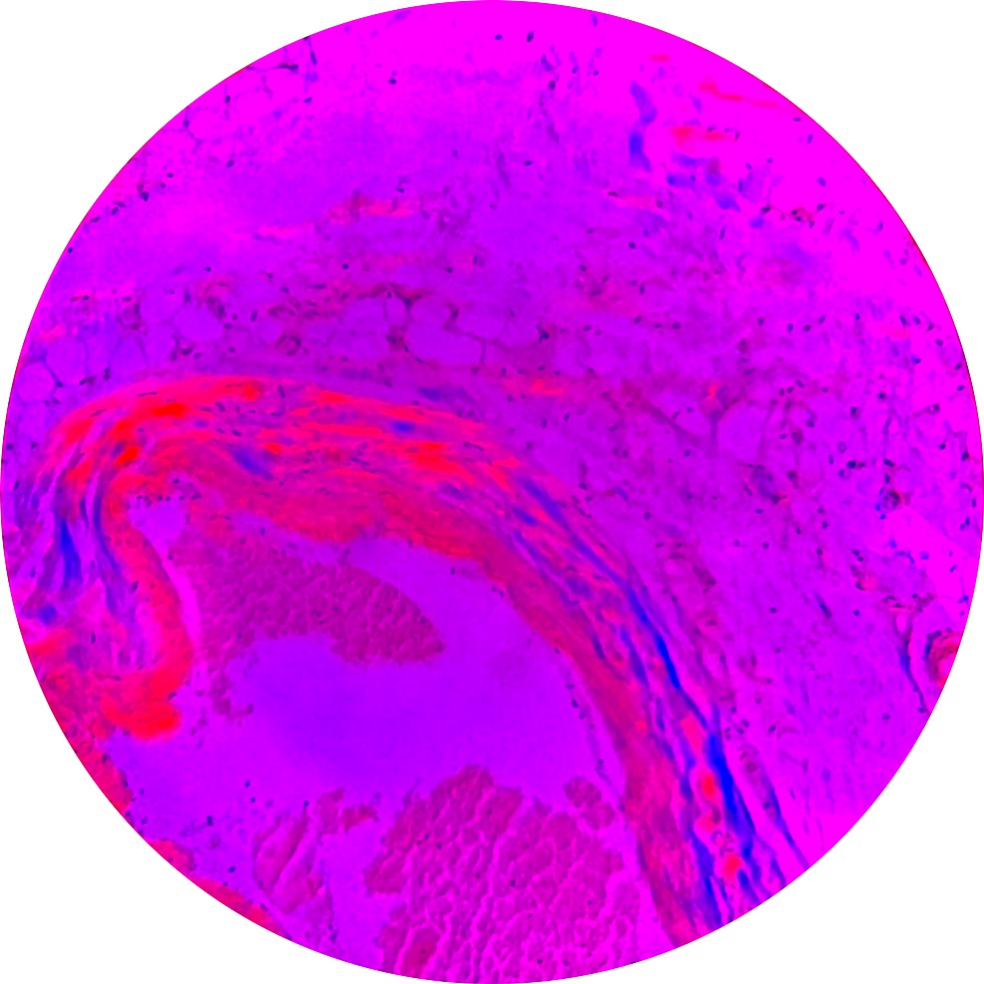
Congo red stain-positive amyloid areas in bright pink

Video-assisted thoracoscopic surgery with the creation of a pericardial window and pleural and pericardial biopsies revealed homogeneous deposits within the walls (Figure 3) consistent with AL amyloid, which was confirmed by Congo stain and polarized light (Figure 4). Serum protein electrophoresis (SPEP) showed an abnormal band in the gamma region, detected equal to 0.04 g/dL, and serum immunofixation showed free kappa of 4.58 mg/dl and kappa/lambda ratio of 0.06. A biopsy was planned but the patient was unable to be weaned off ventilator support, suffered cardiac arrest, and died.

## Discussion

The true incidence and prevalence of cardiac amyloidosis are unknown, as it remains largely underdiagnosed. In recent studies, disease incidence is estimated to be around 17 per 100,000 patients in people above 65 years of age [5]. Cardiac amyloidosis can be classified into the following types: AL (immunoglobulin light chain) amyloidosis (previously referred to as primary amyloidosis), amyloid A (AA) amyloidosis (previously referred to as secondary amyloidosis), isolated atrial amyloidosis (IAA), and transthyretin amyloidosis (ATTR). Of these, AL and ATTR types are the most common.

AL amyloidosis is a systemic, acquired disorder of plasma cells that may present with or without coexisting multiple myeloma. In around 70% of patients, either the heart and/or the kidneys are involved [6]. AL light chain deposits have both infiltrative and directly cardiotoxic effects, and cardiac involvement is associated with high mortality. An increase in reactive oxygen species and up-regulation of heme oxygenase resulting in impairment in contractility and relaxation are likely to play a role on a cellular level [2].

Cardiac amyloidosis may present as congestive heart failure (dyspnea on exertion, orthopnea, hypotension, fatigue, weakness, ascites, and peripheral edema), conduction abnormalities (atrial fibrillation and second- or third-degree heart block), pleural or pericardial effusions, and/or sudden cardiac arrest. Extra-cardiac signs and symptoms may include macroglossia, purpura, petechiae or ecchymosis, sensory neuropathy, nephrotic syndrome, diarrhea, weight loss, or carpal tunnel syndrome [7].

Very few cases of cardiac amyloidosis-induced chylothorax have been described in the literature [3,8-10]. Chylothorax is the extravasation of “chyle,” a milky fluid consisting of lymph and fat droplets, into the pleural space, and it is diagnosed by demonstrating a pleural fluid triglyceride concentration above 110 mg/dl in the correct clinical context, and/or detection of chylomicrons on pleural fluid lipoprotein electrophoresis [11]. Causes of chylothorax can be divided into traumatic, including iatrogenic injury during surgery, and nontraumatic, such as neoplasms, particularly lymphomas, liver cirrhosis, superior vena cava thrombosis, nephritic syndrome, heart failure, and tuberculosis, among others [8].

AL amyloidosis can occur at any stage of plasma cell dyscrasia. Useful laboratory tests include SPEP, urine protein electrophoresis (UPEP), immunofixation electrophoresis, and serum free light chain ratio [3]. N-terminal pro-B-type natriuretic peptide (NT-proBNP) is a sensitive marker of cardiac toxicity and levels greater than 152 pmol/L are associated with a worse prognosis.

An echocardiogram is the initial test of choice in suspected cardiac amyloidosis, and the hallmark feature is increased left ventricular thickness. Other findings include left atrial enlargement and diastolic and/or systolic dysfunction. Another important feature that helps distinguish cardiac amyloidosis from other causes of left ventricular hypertrophy (LVH) is the global longitudinal strain (GLS) pattern, which is both sensitive and specific for cardiac amyloidosis [12]. Late gadolinium enhancement (LGE) on cardiac magnetic resonance (CMR) is also diagnostic and has prognostic significance [13]. Other imaging modalities include bone scintigraphy and single photon emission computed tomography (SPECT-CT). A definitive diagnosis can be made by identifying Congo red-positive extracellular deposits that show apple-green birefringence on biopsies from extra-cardiac sites like the abdominal fat pad or the rectum obtained by fine-needle aspiration [3,7].

The two main goals of therapy in cardiac amyloidosis are to eliminate the immunoglobulin light chain-producing plasma cells and to provide optimal symptomatic care for both the disease and the side effects of therapy [14]. Salt restriction and loop diuretics are the mainstays for the management of heart failure symptoms. However, despite their use in the management of systolic heart failure, angiotensin-converting enzyme inhibitors and β-blockers have failed to demonstrate any prognostic benefit and are usually poorly tolerated, especially in AL amyloidosis, as they can worsen renal function or lead to postural hypotension. Calcium channel blockers can worsen left ventricular function and are also usually avoided [15]. Lastly, digoxin has been historically absolutely contraindicated in these patients, after it was found to have increased affinity to cardiac muscle containing amyloid fibrils in vitro, which would presumably lead to an increase in its toxicity. However, recent studies have found that cautious use with lower initial doses and frequent monitoring of drug levels, creatinine, and electrolytes is usually tolerated and may be considered [16,17].

Treatment of the underlying protein misfolding disorder usually involves chemotherapy and/or autologous hematopoietic cell transplantation. At the time of writing, the most common initial chemotherapeutic regimens are bortezomib-based regimens such as bortezomib, dexamethasone, and cyclophosphamide (CyBorD) or melphalan (BMDex) [18]. Furthermore, in a recent clinical trial, the addition of daratumumab, an anti-CD38 antibody, revealed deeper and quicker hematologic responses [19].

Early diagnosis and management are of utmost importance in AL cardiac amyloidosis as the median survival for untreated patients is only around six months from the onset of heart failure [20]. Enormous advancements have been made over the last decade, both in the diagnosis and treatment of this condition, and when these strategies are employed in a timely fashion, they can induce prolonged remission and extend life by many years. Delayed recognition is frequently associated with poor treatment outcomes, which is readily illustrated in the case presented. By the time of diagnosis, our patient had AL amyloidosis-induced advanced cardiac dysfunction, which combined with her multiple comorbidities, resulted in her passing within less than six months of presentation.

## Conclusions

Cardiac amyloidosis is a rare disease that can present as heart failure, angina, arrhythmias, and pleural or pericardial effusions, among others. In extremely unusual cases, it can also lead to chylothorax. Significant advancements in diagnosis and therapy have been made in the last decade, leading to an improvement in prognosis when the disease is recognized in its early stages. This further emphasizes that physician awareness of this condition is crucial for improved outcomes.
